# Advanced Research in the Pathophysiology of Venous Thromboembolism–Acute Pulmonary Embolism

**DOI:** 10.3390/biomedicines13040906

**Published:** 2025-04-08

**Authors:** Anna M. Imiela, Joanna Kucharska, Franciszek Kukliński, Teresa Fernandez Moreno, Konrad Dzik, Piotr Pruszczyk

**Affiliations:** 1 Department of Internal Diseases and Cardiology, Infant Jesus Clinical Hospital, Medical University of Warsaw, Lindleya 4 Street, 02-005 Warsaw, Poland; 2 Department of Intensive Cardiac Care, Medical University of Bialystok, 15-089 Białystok, Poland

**Keywords:** cytokines, endothelium, inflammation, immunity, pulmonary embolism, thromboinflammation

## Abstract

According to the literature, cardiovascular diseases (CVDs)—including myocardial infarction, stroke, and venous thromboembolism (VTE)—are among the leading causes of mortality and morbidity worldwide. Evidence suggests that CVDs share common risk factors and pathophysiological mechanisms. Similar to the Mosaic Theory of Hypertension proposed by Irvine Page in 1949, the pathophysiology of VTE is multifactorial, involving multiple interacting processes. The concept of immunothrombosis, introduced by Engelmann and Massberg in 2009, describes the interplay between the immune system and thrombosis. Both thrombosis and hemostasis share core mechanisms, including platelet activation and fibrin formation. Additionally, immune mediators—such as monocytes, neutrophil extracellular traps (NETs), lymphocytes, selectins, and various molecular factors—play a critical role in thrombus formation. This review highlights inflammation as a key risk factor for pulmonary embolism (APE). Immunity is central to the complex interactions among the coagulation cascade, platelets, endothelium, reactive oxygen species (ROS), and genetic factors. Specifically, we examine the roles of the endothelium, immune cells, and microRNAs (miRNAs) in the pathophysiology of APE and explore potential therapeutic targets. This review aims to elucidate the roles of the endothelium, immune cells, and miRNAs in the pathophysiology of APE and explore potential future perspective.

## 1. Introduction

Over the past several years, accumulating evidence has demonstrated the intricate interplay between immunity and thrombosis. The concept of immunothrombosis was first introduced in the 1970s by Engelmann and Massberg [1,2]. Traditionally, thrombosis is understood as the occlusion of blood vessels, restricting blood flow with all its consequences. It is also considered a cause and a pathological form of vascular repair in response to local hemostasis. Both thrombosis and hemostasis share common core mechanisms: platelet activation and fibrin formation [1]. Additionally, immune cell mediators—such as monocytes, neutrophil extracellular traps (NETs), lymphocytes, selectins, and various molecular factors—play a crucial role in thrombosis. Immunothrombosis primarily occurs in the microvessels, where fibrin, NETs, monocytes, and platelets interact to recognize and eliminate pathogens [3].

**Figure 1:**
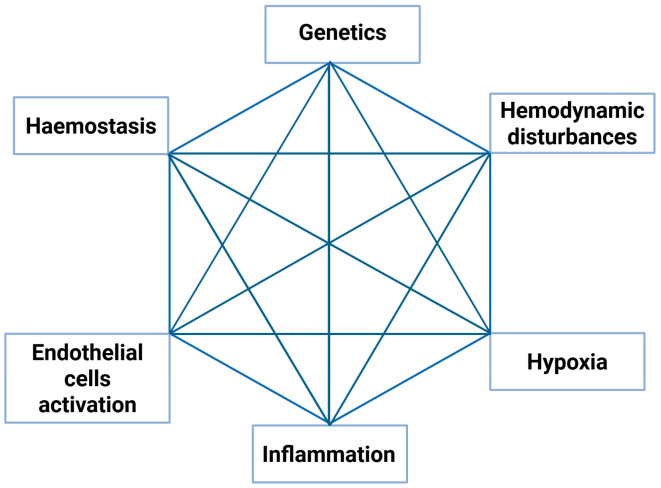
A complex interplay between immunity and other essential factors in the pathophysiology of pulmonary embolism—a mosaic theory of acute pulmonary embolism [8]. The figure was created with the usage of BioRender software (https://www.biorender.com/).

According to the literature, cardiovascular diseases (CVD), including myocardial infarction, stroke, and venous thromboembolism (VTE), are among the leading causes of mortality and morbidity worldwide [4,5,6]. It has been postulated that CVDs share common risk factors and pathophysiological mechanisms. Similarly to the Mosaic Theory of Hypertension proposed by Irvine Page in 1949, the pathophysiology of VTE is also multifactorial [7]. Our previous research introduced a revised Mosaic Theory of Acute Pulmonary Embolism (APE), highlighting immunity as a crucial risk factor for APE *(*Figure 1) [8,9]. Inflammation plays a central role in the complex crosstalk among the coagulation cascade, platelets, endothelium, reactive oxygen species (ROS), and genetic factors.

The Canakinumab Anti-Inflammatory Thrombosis Outcomes Study (CANTOS) was a groundbreaking clinical trial that investigated whether targeted anti-inflammatory therapy, specifically the interleukin-1β (IL-1β) inhibitor canakinumab, could reduce cardiovascular events in high-risk patients [10]. While CANTOS (IL-1β inhibition) confirmed the inflammatory hypothesis of atherosclerosis, the COLCOT trial demonstrated that a widely available drug (colchicine) could achieve similar cardiovascular benefits with fewer risks. Low-dose colchicine significantly reduces the risk of recurrent cardiovascular events in post-MI patients [11]. With its low cost, established safety profile, and strong efficacy, colchicine has emerged as a promising anti-inflammatory therapy for secondary cardiovascular prevention. As inflammation continues to be recognized as a major driver of cardiovascular disease, colchicine may become a key component of standard post-MI care, alongside statins, antiplatelets, and other secondary prevention strategies.

The aim of this review is to focus on the interplay between immunity and thrombosis in APE. In addition, it aims to elucidate the roles of the immune cells, NETs, cytokines, endothelium, and miRNAs in the pathophysiology of APE and to provide new insights into potential therapeutic targets.

## 2. The Relationship Between Inflammation and Acute Pulmonary Embolism in Animal Models

The first studies using experimental models of APE have demonstrated that innate immunity plays a crucial role in the early stages of the disease. In one of the earliest APE models, where thrombosis was induced in the inferior vena cava (IVC) of Sprague–Dawley (SD) rats, Eagleton et al. observed the early infiltration of neutrophils and monocytes in the pulmonary artery (PA) wall in APE-affected rats compared to controls [12]. Moreover, a significantly higher concentration of monocyte chemoattractant protein-1 (MCP-1) was observed in the vessel wall, indicating hyperplasia [12]. MCP-1 exerts chemoattractant effects by recruiting monocytes. Traditionally, neutrophil levels peak within two days after the onset of APE, whereas macrophages increase as early as the first day [12]. Interestingly, an exaggerated influx of macrophages was observed in the lung parenchyma [12].

In another experimental APE model, induced by the accumulation of polystyrene microspheres, a significant increase in neutrophils was observed in the bronchoalveolar lavage (BAL) [13]. Additionally, the neutrophil concentration correlated with APE severity. Rats with higher right ventricular systolic pressure (RVSP) exhibited greater neutrophil accumulation, higher chemoattractant activity, and elevated levels of macrophage inflammatory protein-1 (MIP-1) and cytokine-induced neutrophil chemoattractant-1 (CINC-1) in the BAL, compared to both controls and APE rats with moderate RVSP (35 mmHg) [13]. Similarly, the inflammatory state in the right ventricular (RV) muscle contributed to heart failure and RV dysfunction [14]. The severity of RV dysfunction was associated with higher levels of monocyte chemoattractant protein-1 (MCP-1) and increased myeloperoxidase (MPO) activity, compared to both controls and rats with lower estimated RVSP [14]. Moreover, neutrophils and macrophages were detected in necrotic cardiomyocytes in rats with pulmonary hypertension [14]. However, treatment with anti-polymorphonuclear (anti-PMN) antibodies was associated with a significant reduction in cardiac tissue damage and a decrease in MPO levels in cardiomyocytes [14]. CINC-1 and CINC-2 were found to be responsible for excessive neutrophil recruitment, particularly on the second day after an APE episode [15]. However, treatment with anti-CINC antibodies effectively diminished the neutrophil influx in RV muscle [15].

As a continuation, Watt et al. demonstrated that the intensive influx of neutrophils is not only present at the early stages of an APE episode but it can last even up to 4 weeks [16]. However, macrophages M1 form a dominant cell phenotype after APE at the first day during an APE episode; however, the M2 phenotype induces scar formation together with the myofibroblasts [16].

A series of chemokine gene expression was also assessed. Zagorski et al. demonstrated the upregulation of crucial chemokines for recruiting T lymphocytes (MIP-1, IP-10, MCP-1) and neutrophils (MIP-2 and CINC-1,2] [13]. In addition, the mRNA expression of these chemokines were also upregulated in RV cardiomyocytes in rats with severe APE and higher estimated RVSP as compared with rats with only mild pulmonary hypertension [14]. Even with a moderate APE severity (with RVSP around 40 mmHg), the receptors CCR1 and CXCR4 were upregulated [17]. Next, transcriptional changes were also observed depending on the RV anatomy. Surprisingly, numerous pro-inflammatory and pro-fibrotic pathways (such as fibroblasts growth factors, connective tissue growth factor, collagen, and cysteine-rich protein-61) were upregulated in the RV outflow tract (RVOT) in comparison with the RV apex [18]. Six weeks after the APE episode, there is a transcriptome shift, the upregulation of matrix metalloproteinases in RVOT and downregulation of key genes responsible for cell metabolism (fatty acids, amino acids, and carbohydrates) [18]. According to a study using the rabbit model of APE with the usage of an autologous clot, the upregulation of genes was mainly related to inflammatory proteins, such as IL-8, TNF-α, Toll-like receptors (TLR), T and B cell receptor signaling pathways, and nucleotide binding and oligomerization domain-like signaling pathways [19]. It can be postulated that transcriptome changes are more exaggerated in the lung parenchyma than in the RV muscle. Eighteen hours after the APE episode, there were no significant changes in cardiomyocytes, while in the lung parenchyma, significant alterations were observed [20]. Even short-lasting, an APE episode of two hours with mildly elevated RVSP (around 44 mmHg) was associated with severe transcriptome changes in the lung parenchyma [20].

**Figure 2:**
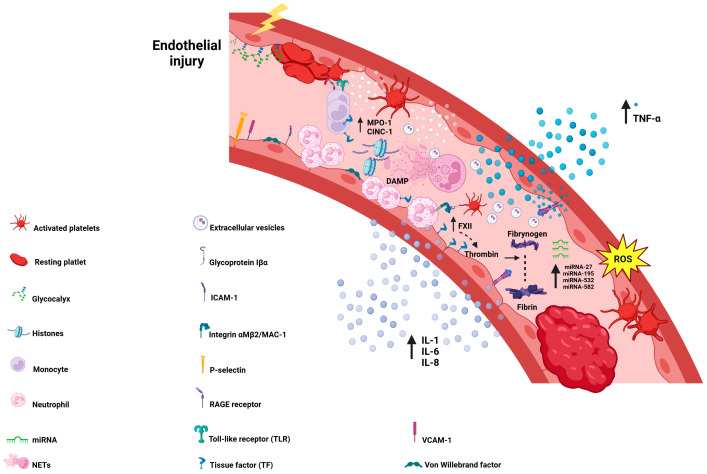
Primarily, the process of thromboinflammation is triggered by the release of danger-associated molecular patterns (DAMPs) in the bloodstream following cell injury and exaggerated adhesive molecule expression on the endothelial cell surface (ICAM-1,VICAM-1,P-selectin). DAMPs interact with the endothelium and promote the release of cytokines and chemokines. Next, endothelial dysfunction, characterized by platelet and leukocyte recruitment, will in turn become activated and secrete pro-inflammatory and pro-coagulant molecules, further contributing to thrombosis. Activated platelets and endothelial cells induce NET formation and TF expression in monocytes. This initiates the coagulation cascade through both intrinsic and extrinsic pathways and finally leads to thrombin-induced fibrin generation and thrombus burden augmentation. Abbreviations: CINC-Cytokine-induced neutrophil chemoattractant 1; DAMP-Danger associated molecular pattern; ICAM-Intercellular Adhesion Molecule; IL-Interleukin; MPO-Myeloperoxidase; NETs-Neutrophil traps; RAGE-Receptor for Advanced Glycation End-products; ROS-reactive oxygen species; TF-Tissue factor; TLR-Toll like receptor; TNF-Tumor necrosis factor.

In general, in the early hours and days after an APE episode, the components of innate immunity, such as macrophages, monocytes, and neutrophils, play a major role in the pathophysiology of the inflammatory reaction (Figure 2). Circulating immune cells attach to the endothelium and contribute to the pro-inflammatory milieu through the secretion of cytokines and chemokines. Traditionally, monocytes are known as phagocytic cells acting as major players in the innate immunity system in VTE [21]. Monocytes, being a major group of cells dominating the early phase, express active tissue factor (TF) and weaken thrombus resolution, resulting in clot stabilization [22,23]. Moreover, monocytes recruit platelets to the site of the inflammation and promote coagulation and inflammasome activation [24]. In experimental models of VTE, the complete elimination of monocytes with the usage of clodronate in the FeCl_3_ model was associated with a reduction in the DVT burden [25]. Parallelly, the role of granulocytes in thrombus formation is inevitable (Figure 2) [26]. In an animal model of DVT based on eosinophil-deficient mice, eosinophils were capable of impairing thrombus generation by reducing TF expression [26]. Interestingly, the most numerous cells in the venous thrombi remained neutrophils. In a series of studies, it has been demonstrated that neutrophils are engaged not only in thrombus formation but also in fibrinolysis and clot resolution [23].

In summary, the experimental models of APE have significantly advanced our understanding of the innate immune system’s role in the early pathophysiology of the disease. Key findings from these models indicate that neutrophils, monocytes, and macrophages are primary drivers of the inflammatory response, tissue remodeling, and immune activation following an APE episode. The upregulation of chemokines, cytokines, and transcriptional changes in both the pulmonary vasculature and right ventricular (RV) muscle further highlights the profound immunological and inflammatory shifts associated with APE. Furthermore, transcriptomic studies reveal significant genetic alterations in both the RV muscle and lung tissue, with the upregulation of inflammatory signaling pathways, fibroblast growth factors, and matrix metalloproteinases, particularly in the RV outflow tract (RVOT). Notably, even short episodes of APE with mild elevations in RV systolic pressure lead to widespread transcriptomic shifts in lung tissue, emphasizing the immunological sensitivity of the pulmonary environment to thromboembolic events.

Additionally, innate immune cells play a dual role in thrombus formation and resolution. Monocytes and neutrophils interact with platelets, express tissue factor (TF), and activate inflammasome pathways, promoting clot stabilization. Conversely, neutrophils are also implicated in fibrinolysis and clot resolution, underscoring their complex role in venous thromboembolism (VTE) pathology.

Overall, these findings suggest that targeting innate immune pathways could be a promising therapeutic approach for mitigating inflammatory damage, preventing RV dysfunction, and improving thrombus resolution in APE. Future research should focus on refining anti-inflammatory strategies, such as selective chemokine inhibitors or immune cell modulation, to optimize clinical outcomes in APE patients.

## 3. The Interplay Between Thrombosis and Neutrophils and Neutrophil Extracellular Traps (NETs)

Neutrophils constitute 50–70% of all leucocytes and are the major granulocyte subpopulation. Neutrophils circulate in the bloodstream; however, under inflammatory conditions, they can migrate from the systemic circulation into inflamed tissue and inactivate microbes. Neutrophils have developed numerous strategies, such as phagocytosis to eliminate pathogens; however, the most essential is the ability to deploy their contents to the extracellular space. This phenomenon, known as NETosis, was first described in 2004 by Brinkmann et al., and its principal aim is the destruction of bacteria and other pathogens by releasing traps composed of decondensed chromatin fibers, histones, and granule components [27]. NETs contain DNA, histones, and enzymes that trap pathogens. NET formation (NETosis) results in the release of histones, which promote thrombosis. Additionally, NETs act as a scaffold for thrombi and impair fibrinolysis, enhance platelet activation, and interact with coagulation factors (Figure 2) [21,28].

### 3.1. Data Summarizing the Role of NETs in Animal Studies

A growing body of evidence has demonstrated that NET formation is closely related to VTE. In the study performed by von Bruhl et al., in murine hypoxia-induced DVT, neutropenia or the disintegration of NETs, as well as the genetic ablation of FXII, act as protective factors and ameliorate thrombus formation and amplification [24]. Next, the inhibition of a crucial enzyme for NET activation, peptidyl deiminase 4 (PAD-4), with the usage of PAD-4 inhibitors, has been associated with the disruption of NET formation and thrombus generation [29].

The impact of NETosis on thrombosis is multidimensional. First, the neutrophil–platelet interaction mediated by integrin CD11b/CD18, known also as Mac-1 (at the surface of neutrophils) and glycoprotein Ibα (at the surface of platelets), contributes to thrombosis mediated by platelet activation and accumulation (Figure 2) [23,30]. The initiation of the intrinsic coagulation pathway occurs through the direct activation of factor XII (FXII), leading to thrombin generation and clot formation. Activated platelets contribute to the contact coagulation pathway by releasing polyphosphate (polyP), while also initiating the extrinsic coagulation pathway through TF activation

Moreover, DNA-bound histones released during NETosis exhibit prothrombotic activities by stimulating the endothelium to produce and secrete von Willebrand factor (VWF) [23,31]. Histones, cationic nuclear proteins responsible for packaging DNA into nucleosomes, can act as damage-associated molecular patterns (DAMPs) when released into the extracellular space, thereby triggering immune responses (Figure 2) [32].

Experimental studies have demonstrated that histones induce microvascular thrombosis when injected into mice. They contribute to clot formation by directly interacting with fibrinogen, prothrombin, and VWF. The protein C–thrombomodulin (TM) system plays a key regulatory role in coagulation by inhibiting thrombin generation. Specifically, thrombin bound to TM on endothelial cells activates protein C, which subsequently degrades clotting factors Va and VIIIa, thereby reducing clot formation [32]. However, histones impair this anticoagulant pathway by binding to both TM and protein C, thereby preventing protein C activation. This disruption results in excessive thrombin production and uncontrolled thrombosis. Additionally, higher concentrations of VWF and its interaction with DNA–histone complexes lead to exaggerated platelet accumulation [23,31,33]. Interestingly, DNA–histone complexes also promote a procoagulant state by inhibiting protein C activation through the thrombin–thrombomodulin complex [32]. Additionally, NETs release proteases that degrade tissue factor pathway inhibitor (TFPI), thereby reducing its anticoagulant potential [34]. Neutrophil elastase (NE) and MPO further contribute to thrombosis by degrading anticoagulant proteins, such as TFPI and TM, while also limiting the fibrinolytic activity of tissue plasminogen activator (tPA) [35].

The immune system’s contribution to creating a pro-coagulant environment is referred to as immunothrombosis. This process, which plays a crucial role in the body’s natural defense against pathogens, involves the formation and stabilization of thrombi through immune-mediated mechanisms. However, when immunothrombosis becomes dysregulated—especially in conjunction with endothelial dysfunction—it can exacerbate sterile inflammation and promote venous thrombosis.

### 3.2. Data Demonstrating the Role of NETs in Thromboembolism in Humans

NETs play a role in acute thrombotic conditions, as shown by elevated MPO–DNA levels in ATE patients. However, NET markers do not predict the long-term cardiovascular risk. The study performed by Bressan A demonstrated the connection between NET formation and different acute thrombotic conditions, including acute coronary syndrome (ACS) (*n* = 60), cerebrovascular accident (CVA) (*n* = 50), and venous thromboembolism (VTE) (*n* = 55). A total of 165 patients with ATE were compared to 70 control patients (admitted for chest pain but with no ATE). MPO–DNA complexes were significantly elevated in ATE patients compared to controls (*p* < 0.001). This remained statistically significant even after adjusting for traditional cardiovascular risk factors (*p* = 0.001). An ROC analysis showed that MPO–DNA complexes had an AUC of 0.76 (95% CI: 0.69–0.82), suggesting moderate diagnostic power in distinguishing ATE patients from controls. However, NET markers do not predict long-term cardiovascular risk. Future studies should explore NET-targeted therapies and refine the clinical utility of NET biomarkers.

The study conducted by Smith P et al. aimed to evaluate NET-related markers (neutrophil elastase (NE), nucleosomal citrullinated histone H3 (H3Cit-DNA), and cell-free DNA) and compare their diagnostic accuracy to D-dimer [36]. The plasma levels of NE, H3Cit-DNA, and cell-free DNA were measured in 294 patients with suspected VTE (pulmonary embolism and deep vein thrombosis) and compared to 30 healthy controls. Finally, participants included 112 VTE-positive and 182 VTE-negative patients from two prospective cohort studies [36]. Higher levels of NE and H3Cit-DNA were observed in VTE patients. Cell-free DNA was not significantly associated with VTE. D-dimer showed superior diagnostic accuracy (AUC: 0.90 and 0.93) compared to NE (AUC: 0.65 and 0.68) and H3Cit-DNA (AUC: 0.60 and 0.67). Adding NET markers to D-dimer did not improve diagnostic performance [36]. NET formation occurs in VTE, as indicated by increased NE and H3Cit-DNA levels. However, these markers do not outperform D-dimer for the diagnosis. The potential of NET biomarkers for a VTE diagnosis appears to be limited. In addition, in the studies conducted by Ząbczyk M et al., the prothrombotic clot properties and NETosis activation (citH3 increase) were linked to higher early mortality risk in acute PE [3].

## 4. Soluble Cytokines and Chemokines in the Pathophysiology of Acute Pulmonary Embolism—A Brief Summary

Cytokines, chemokines, adhesion molecules, and matrix metalloproteases (MMPs) form critical links between inflammation and coagulation [37]. Cytokines, a heterogeneous group of immunomodulating agents, mediate inflammatory responses and contribute to the development of venous thrombosis. Elevated concentrations of pro-inflammatory cytokines, along with genetic variations affecting cytokine production, have been observed across various patient cohorts. Although these elevations are consistently reported in both animal models and clinical cases, their diagnostic value remains uncertain (Table 1). The interplay among these factors influences thrombus formation, resolution, and the development of post-thrombotic complications [8,37]. Evidence from animal models and clinical studies (Table 1 and Table 2) suggests that the immune system plays a pivotal role not only in thrombus formation but also in predisposing individuals to VTE. Cytokines and chemokines, in particular, have been identified as key contributors to this process (Figure 2).

The major pro-inflammatory cytokines involved in VTE pathogenesis are tumor necrosis factor-alpha (TNF-α), interferon, interleukin-1 (IL-1), interleukin-6 (IL-6), and interleukin-17 (IL-17). Elevated levels of these cytokines have been consistently observed in experimental models of VTE (Table 1). The roles of specific cytokines have been described in detail elsewhere [8,9]. Briefly, the IL-1 cytokine family plays a crucial role in inflammation, linking it to coagulation and fibrinolysis—key processes in VT development [37,43]. Genetic alterations in cytokine genes, such as IL-1β and IL-6, may increase susceptibility to VTE. However, some single-nucleotide polymorphisms (SNPs) have also been associated with a reduced risk of VTE, highlighting the complexity of these genetic interactions. Elevated pro-inflammatory cytokines, like IL-6 and TNF-α, are commonly detected in patients during acute VTE episodes; however, their diagnostic utility remains limited. Despite promising insights, no inflammatory biomarker currently surpasses traditional markers, such as D-dimer, in clinical practice.

MMPs also play a crucial role in orchestrating the immune response in VTE. Along with their natural inhibitors, tissue inhibitors of metalloproteinases (TIMPs), MMPs regulate vessel wall remodeling and thrombus resolution. Specifically, MMP-9 levels rise during acute thrombosis and decrease upon resolution, suggesting a role in thrombus breakdown. Genetic variations, particularly SNPs affecting MMP-9 and IL-6, have been linked to an increased risk of VTE, especially in cancer patients (Figure 2) [37,44].

The balance between MMPs and TIMPs may determine the extent of vessel wall fibrosis, thereby influencing long-term outcomes, such as post-thrombotic syndrome (PTS) [37]. Numerous experimental studies have demonstrated that MMPs contribute to both vessel wall fibrosis and thrombus resolution [45,46]. In a clinical cohort study involving 201 patients with VTE, higher plasma concentrations of MMPs during the acute phase and at 18 months of follow-up were associated with the development of PTS [47]. The link between inflammation and thrombosis opens new avenues for diagnostics and therapy. However, further research is essential. Future studies should prioritize validating these biomarkers in diverse patient populations, particularly those with concurrent inflammatory diseases. A deeper understanding of genetic predispositions and individual inflammatory responses could pave the way for personalized therapeutic strategies.

**Table 2:** Table summarizing the role of cytokines and chemokines in the pathophysiology of acute pulmonary embolism.

Marker	Author	Year	Study Design	Total Patients	Conclusion
IL-1	Van Minkelen R. [43]	2007	Case-control	948	The H5H5 homozygous genotype of the IL-1RN gene increases the risk of VT.
	Abuduhalike, R. [48]	2020	Prospective	284	The IL-1 SNP-rs1800587 GG + GA variant is associated with higher risk of VTE in comparison to subjects with the AA genotype [OR 4.444, 95% CI 1.466–13.470].
IL-6	Van Aken, B.E. [49]	2000	Case-control	532	Higher plasma concentrations of IL-6 in patients with deep vein thrombosis.
	Roumen-Klappe, E.M. [50]	2002	Prospective	73	
	Roumen-Klappe, E.M. [51]	2009	Prospective	110	
	Beckers, M.M.J. [52]	2010	Prospective	433	
	Matos, M.F. [53]	2011	Prospective	245	
	Bittar, L.F. [54]	2015	Case-control	135	
	De Franciscis, S. [47]	2016	Prospective	201	
	Zhang, Y. [55]	2019	Case-control	72	Lower expression of miR-338-5p contributes to DVT by enhancing IL-6 expression.
IL-8	Van Aken, B.E. [49]	2000	Case-control	532	Venous thrombosis risk is increased in patients with circulating IL-8 levels.
	Van Aken, B.E. [56]	2002	Population-based case-control study	948	
	Roumen-Klappe, E.M. [50]	2002	Prospective cohort	73	
	Montes-Worboys, A. [57]	2013	Follow-up	125	
	Bontekoe, E. [58]	2021	Prospective	157	
	Tang B. [19]	2014	Case-control	660	The IL-10 (-1082A/G) gene polymorphism’s GG genotype has a protective effect, lowering the risk of DVT.
IL-13	Beckers, M.M.J. [52]	2010	Prospective	433	The study identified an association between VTE episodes and SNPs located in the genes encoding IL-13.
TNF-α	Horakova, K. [59]	2010–2011	Case-control	129	Patients with DVT had a higher prevalence of the G-308A polymorphism than the control group.
	Mazetto, B.M. [60]	2012	Prospective	56	Higher plasma concentration of TNF-α in patients with VTE compared to healthy controls.
	De Franciscis, S. [47]	2015	Prospective	201	
TGF-β	Memon, A.A. [61]	2014	Prospective with 39 months follow-up	126	Patients with VTE were characterized by a lower plasma concentration of TGF-β1 and TGF-β2.
	Wang, X. [62]	2019	Prospective	78	The higher risk of VTE reoccurrence was related to 12 mRNAs: miR-15b-5p, miR106a-5p, miR-197-3p, miR-652-3p, miR-361-5p, miR-222-3p, miR-26b-5p, miR-532-5p, miR-27b-3p, miR-21-5p, miR-103a-3p, and miR-30c-5p.
MCP-1	van Aken, B.E. [49]	2000	Case-control	532	Plasma concentrations of MCP-1 were elevated in patients with recurrent venous thrombosis.
	Bontekoe, E. [58]	2021	Prospective	157	A significant increase in the levels of MCP-1 was observed in patients with submassive or low-risk PE.
ICAM-1	Shbaklo, H. [63]	2009	Prospective	387	Patients with PTS had higher ICAM-1 levels compared to those without PTS (ICAM-1: *p* = 0.06).
P-selectin	Vandy, F.C. [64]	2013	Prospective	279	In patients characterized by a Wells score ≥ 2, sP-selectin has shown a 97.5% specificity and a 91% PPV for diagnosing DVT.
	Vandy, F.C. [64]	2013	Prospective	279	In patients characterized by a Wells score ≥ 2, sP-selectin has shown a 97.5% specificity and a 91% PPV for diagnosing DVT.

## 5. Endothelium

### 5.1. Role of the Endothelium in Venous Thromboembolism

Virchow’s triad—comprising hypercoagulability, alterations in blood flow (such as stasis or turbulence), and endothelial damage—remains the cornerstone of VTE pathogenesis, driving thrombus formation and propagation. During VTE, an intricate cascade of cellular signals leads to the excessive recruitment and activation of monocytes, platelets, and neutrophils, culminating in sterile inflammation.

Recent studies have deepened our understanding of these processes, highlighting the pivotal roles of inflammatory and immune factors that contribute to the complex cellular-level pathomechanisms of VTE. Endothelial dysfunction, marked by a prothrombotic and pro-inflammatory state, plays a central role in the pathophysiology of various cardiovascular diseases, including atherosclerosis, stroke, hypertension, and venous thrombosis. By promoting coagulation and disrupting vascular homeostasis, endothelial dysfunction serves as a critical link between inflammation and thrombosis [65,66,67,68].

In recent years, the intricate interplay between thrombosis and inflammation has become increasingly evident. Platelets, inflammatory cells, and endothelial cells mediate these interconnected processes [69]. Under physiological conditions, inflammation activates the coagulation cascade as part of the body’s defense mechanism against pathogens [65]. However, in VTE, an exaggerated inflammatory response leads to the overactivation of monocytes, platelets, and neutrophils, driving the development of sterile inflammation (Figure 2). This form of inflammation, occurring without bacterial or viral infection, is commonly observed in acute conditions, such as trauma or reperfusion injury [65]. A comprehensive understanding of the pathomechanisms underlying thrombus formation is essential for identifying reliable biomarkers of endothelial dysfunction and developing targeted therapies. Such advancements hold the potential to restore endothelial homeostasis and prevent vascular complications, ultimately improving patient outcomes.

### 5.2. Physiological Function of Endothelial Cells

The endothelium, first described in 1865, is a monolayer of cells lining blood vessels that functions as a structural barrier, maintains blood fluidity, and regulates vascular homeostasis through processes, such as angiogenesis, thrombosis, immune responses, and inflammation [8,9,65,70]. Endothelial cells (EDs) play a central role in vascular physiology by facilitating hormone and nutrient transport, regulating vessel permeability and tone, modulating coagulation, and supporting angiogenesis [69,71]. Notably, they can adapt their phenotype in response to changes in the microenvironment, underscoring their dynamic nature [72]. Under physiological conditions, ECs exhibit antithrombotic, anti-inflammatory, and vasodilatory properties [65].

They produce key anticoagulant molecules, including heparin-like proteoglycans (glycocalyx), TFPI, TM, and endothelial protein C receptor (EPCR), all of which contribute to coagulation regulation. The vascular tone is maintained through nitric oxide (NO) production, while prostacyclin I2 (PGI2) inhibits platelet adhesion. Additionally, endothelial-derived fibrinolytic factors—tPA and urokinase plasminogen activator (uPA)—convert plasminogen to plasmin, facilitating clot breakdown [65]. The glycocalyx’s anti-adhesive properties prevent VWF from binding to platelets, a process regulated by ADAMTS13 (a disintegrin and metalloproteinase with thrombospondin type 1 motif, member 13) [65,73]. In murine models of endotoxemia, ADAMTS13 deficiency has been associated with increased leukocyte adhesion, driven by the excessive secretion of WP bodies. However, during endothelial dysfunction, these protective mechanisms are compromised, shifting the endothelium toward a prothrombotic state. This dysfunction is further exacerbated by systemic inflammation, oxidative stress, and hypoxia, which activate endothelial cells and promote hypercoagulability.

### 5.3. Pro-Inflammatory Molecules—Sterile Inflammation and Endothelium Activation

Inflammation and thrombosis are interconnected in a self-amplifying cycle, where inflammation triggers thrombus formation, and thrombosis, in turn, enhances inflammation through the activation of ECs and platelets [1,74]. Under physiological conditions, inflammation plays a critical role in defending against pathogens. However, a similar cascade of signals can be activated by endothelial injury in the absence of infectious agents, leading to sterile inflammation.

A pro-inflammatory state within the endothelial microenvironment induces significant functional changes in endothelial cells. Cytokines, chemokines, ROS, complement components, and PAMPs/DAMPs [75] contribute to severe endothelial dysfunction. Endothelium damage stimulates the release of key pro-inflammatory molecules, including IL-6, C-reactive protein (CRP), IL-1, and TNF-α [76]. TNF-α-induced signaling in the endothelium activates transcription factors, such as nuclear factor kappa B (NF-κB) and activator protein-1 (AP-1), triggering the synthesis of cell adhesion molecules (CAMs), including vascular cell adhesion molecule-1 (VCAM-1), intercellular adhesion molecule-1 (ICAM-1), and E-selectin. These molecules facilitate neutrophil migration and adhesion to the endothelium, promoting a local inflammatory state (Figure 2). This, in turn, sustains a positive feedback loop of cytokine activation, further leukocyte and platelet adhesion, and the amplification of both thrombosis and inflammation [21].

Importantly, there is a close interaction among the endothelium, platelets, monocytes, and neutrophils, which collectively promote a pro-coagulant state [77]. Macrophages and monocytes, distinct populations of immune cells, play a central role in this vicious cycle. Recruited by chemokine (C-C motif) ligand 2 (CCL2), monocytes adhere to the endothelium via E- and P-selectins (Figure 2). Moreover, monocytes contribute to coagulation by releasing microvesicles containing TF, further enhancing thrombus formation [65,78].

Leukocytes play a central role in orchestrating the early phases of the immune response [79]. Subsequently, the activation of endothelial cells leads to the secretion of soluble forms of cell adhesion molecules (CAMs) into the circulation, promoting the recruitment and adhesion of inflammatory cells to the endothelium. The rolling and adhesion of neutrophils are primarily mediated by P-selectin glycoprotein ligand-1 (PSGL-1). P-selectin, the first protein upregulated in activated endothelial cells and platelets, translocates to the cell surface, facilitating neutrophil migration and adhesion through an interaction with PSGL-1 [65,80].

This interaction not only promotes leukocyte recruitment but also initiates thrombus formation by upregulating tissue factor (TF) expression on monocytes. Elevated levels of soluble ICAM-1 (sICAM-1) and soluble VCAM-1 (sVCAM-1) have been identified as biomarkers of endothelial dysfunction [81]. The enhanced expression of VCAM-1 and ICAM-1 favors neutrophil adhesion. Studies have demonstrated that elevated levels of sICAM-1 are associated with an increased risk of recurrent APE [82]. The tight link between endothelial inflammation and dysfunction, leading to enhanced clot formation, is further reflected by the increased expression of various adhesive glycoproteins. E-selectin, another key glycoprotein involved in thrombosis, is upregulated in activated endothelial cells during inflammation (Figure 2). Unlike P-selectin, E-selectin levels rise later in the thrombus-formation process. Both P-selectin and E-selectin facilitate the recruitment of neutrophils and platelets to sites of inflammation and injury [83].

L-selectin, an adhesion molecule predominantly expressed on the surface of leukocytes—including T cells, B cells, neutrophils, and monocytes—also plays a crucial role in thromboinflammation. Its upregulation during inflammation enhances the migration of immune cells across the endothelium. Recent research has shown that circulating levels of L-selectin are inversely associated with mortality in patients with APE. During thromboinflammation and immune cell activation, free circulating levels of L-selectin decrease as adhesion to endothelial cells increases [84]. Both sICAM-1 and E-selectin serve as sensitive biomarkers of endothelial dysfunction. Recent studies have demonstrated that low concentrations of E-selectin combined with high levels of sICAM-1 are associated with an increased risk of recurrent VTE [82].

Additionally, under pro-inflammatory and pro-coagulant conditions, both platelets and endothelial cells release their contents into the extracellular matrix [85]. The interaction between platelets and fibrinogen via the GPIIb/IIIa receptor sustains endothelial activation. In response to inflammation, endothelial cells release WPBs containing VWF, along with pro-inflammatory chemokines and P-selectin. The GPIbα receptor on the platelet surface interacts with VWF and the endothelium, leading to robust platelet adhesion and accumulation [65,86].

The critical role of the VWF–platelet interaction in the pathogenesis of VTE has been demonstrated in experimental studies. In a murine model of VTE, generated by significant flow restriction in the IVC, Brill et al. observed that VWF-deficient mice did not develop thrombosis [86]. Moreover, mice with only half-normal VWF levels were similarly protected from VTE in the stenosis model. Interestingly, multiple injections of recombinant factor VIII failed to induce thrombosis in VWF-deficient mice, suggesting that impaired coagulation was not the primary reason for the absence of DVT in these animals [86]. These findings highlight the essential role of VWF–platelet interactions in thrombus formation, particularly in flow-disturbance-induced venous thrombosis [86].

To summarize, inflammation and thrombosis form a self-amplifying cycle where inflammation triggers clot formation and thrombosis enhances inflammation. Sterile inflammation, caused by endothelial injury, leads to endothelial dysfunction via cytokines (IL-6, TNF-α, CRP) and the activation of NF-κB, increasing adhesion molecule (VCAM-1, ICAM-1, E-selectin) expression. This promotes neutrophil and platelet adhesion, sustaining inflammation. Monocytes and macrophages further drive coagulation by releasing tissue factor (TF). Biomarkers like sICAM-1 and VCAM-1 indicate endothelial dysfunction and thrombotic risk. Platelets and endothelial cells release von Willebrand factor (VWF), which is critical in clot formation. Experimental studies have confirmed VWF’s essential role in venous thromboembolism (VTE), linking inflammation directly to thrombosis progression.

## 6. Reactive Oxygen Species and High-Mobility Group Box 1 Protein—Vigorous Players in Endothelium-Related Inflammation

Moreover, ROS play a pivotal role in the pathogenesis of VTE and APE by mediating cytokine signaling and facilitating interactions between the inflammatory state and the endothelial cell surface. The primary sources of ROS include eNOS, NADPH oxidase, and mitochondria. Under physiological conditions, eNOS performs anti-inflammatory functions and reduces platelet–endothelium interactions by producing NO. However, under hypoxic conditions, ROS production is markedly increased. This heightened ROS generation is associated with the enhanced formation of NETs, which, in turn, triggers the excessive production of chemokines and cytokines. Additionally, ROS promote a pro-coagulant state by recruiting monocytes and inducing TF expression (Figure 2) [87,88].

Mitochondria, double-membrane organelles responsible for generating adenosine triphosphate (ATP) through aerobic respiration, play a critical role in ROS formation within endothelial cells. A key mechanism involves the release of mitochondrial DNA (mtDNA), which acts as a DAMP, thereby inducing inflammation [65,89]. A vicious cycle is triggered when ROS production activates the NLRP3 inflammasome, which in turn stimulates further ROS generation and promotes the secretion of pro-inflammatory interleukins, including IL-1β and IL-18. This cycle amplifies the inflammatory response, contributing to a prothrombotic state. Additionally, the production of high-mobility group box 1 (HMGB1) is significantly increased. HMGB1, a non-histone DNA-binding protein primarily involved in DNA transcription and replication, also functions as a potent DAMP. It is released predominantly by activated platelets and macrophages [65,90,91]. Platelet-derived HMGB1 interacts with monocytes and neutrophils, triggering a prothrombotic inflammatory response and promoting NET formation [92].

Notably, platelets are the primary source of circulating HMGB1, underscoring their central role in thrombus development. HMGB1 accumulation within clots suggests a direct role in thrombosis progression. In particular, the disulfide isoform of HMGB1 interacts with the endothelium via the receptor for advanced glycation end products (RAGE). This interaction recruits leukocytes and induces the excessive production of adhesion molecules (ICAM-1, VCAM-1), cytokines (IL-6, TNF-α), and chemokines (CCL3, CCL4, CXCL12), further amplifying the prothrombotic inflammatory cascade.

HMGB1 is thus recognized as a central mediator of sterile inflammation—a form of inflammation occurring without infection—and plays a critical role in the prothrombotic cascade. Evidence from murine models supports this role: in a study using *Hmgb1*^−^/^−^ chimeric mice, the transfer of platelets from wild-type mice restored clot formation, confirming the essential contribution of platelet-derived HMGB1 [92]. Targeting specific HMGB1 isoforms presents a promising dual anti-inflammatory and anticoagulant strategy for treating VTE. A study by Dyer et al. demonstrated that HMGB1 deficiency in platelets and megakaryocytes was associated with a reduced thrombus size and diminished NET formation [93,94]. Potential HMGB1-targeted therapeutic strategies include HMGB1 inhibition to mitigate its prothrombotic effects and the usage of oxidation blockers [92,95]. These approaches may disrupt the prothrombotic cascade and offer new avenues for preventing DVT and related vascular complications.

## 7. Altered Phenotype of Endothelial Cells—Coming Back to the Embryogenesis

Recurrent endothelial stimulation triggered by inflammatory conditions, ROS production, excessive chemokine and cytokine secretion, and the release of DAMPs and PAMPs from injured cells promotes endothelial-to-mesenchymal transition (EndMT) [96]. EndMT is a biological process in which endothelial cells undergo phenotypic transformation into mesenchymal-like cells, such as myofibroblasts and smooth muscle cells. This transition involves the loss of endothelial markers, including CD31 and VE-cadherin, accompanied by the acquisition of mesenchymal markers, such as α-smooth muscle actin (α-SMA) and SM22α [97]. The mesenchymal phenotype is associated with enhanced pro-coagulant and pro-inflammatory properties due to increased leukocyte recruitment and elevated production of extracellular matrix components, including collagen and fibronectin [98]. EndMT plays a critical role in embryonic development, particularly in cardiac valve formation. However, accumulating evidence links EndMT to the pathogenesis of numerous CVDs [97,99,100,101]. In pulmonary arterial hypertension (PAH), EndMT is associated with endothelial injury and vascular remodeling [65,97,102]. Similarly, in atherosclerosis, EndMT contributes to plaque formation and instability, compromising endothelial integrity [96,97,103]. Furthermore, the persistence of EndMT in adult heart valves may disrupt valvular interstitial cell turnover, potentially leading to calcific aortic valve disease [65,104].

The EndMT process is modulated by various molecular mechanisms, such as epigenetic modifications, such as DNA and histone methylation [65]. Moreover, microRNAs (miRNAs), for example, miR-20a, miR-21, and miR-200a, regulate EndMT by modulating the TGF-β signaling pathway, either facilitating or inhibiting the process [105]. Long Non-Coding RNAs (lncRNAs), including lncRNAs, such as GATA6-AS, influence EndMT through chromatin remodeling, thereby affecting gene expression [106]. Further, Circular RNAs (circRNAs) play an emerging role, and evidence suggests that circRNAs may act as potential regulators of EndMT, although their exact roles remain under investigation [107]. EndMT represents a fundamental biological process with significant implications for the pathophysiology of the cardiovascular system. However, the absence of a precise molecular definition of EndMT complicates research and standardization. To fully understand its adaptive or pathological roles in the vasculature and translate these findings into clinical applications, further clinical data are required. There is an urgent need for comprehensive transcriptomic and proteomic profiling to elucidate the dynamics of EndMT. Targeting EndMT-related pathways, such as epigenetic regulators or TGF-β signaling inhibitors, holds promise for developing novel therapeutic strategies for CVDs.

## 8. Extracellular RNA—A Link Between the Endothelium and Immunity

Emerging evidence highlights extracellular RNA (eRNA) as a critical cofactor in blood coagulation and immune regulation. During vascular wall injury, eRNA is released from damaged or necrotic cells, positioning it as a DAMP. Acting as a DAMP, eRNA directly influences thrombus formation and progression by activating immune responses [65,108,109]. Endothelial cells release eRNA upon injury, which subsequently binds to TLR3 on endothelial cells, triggering a pro-inflammatory response and the early influx of leukocytes (Figure 2). TLR3 recognizes RNA fragments, including self-RNA, and its activation leads to the production of pro-inflammatory cytokines and chemokines, such as CXCL5 [110].

In an experimental VTE model conducted by Najem MY et al., eRNA was shown to contribute to excessive neutrophil accumulation, the formation of NETs, enhanced CXCL5 secretion, and thrombus formation [76]. The activation of TLR3 by eRNA induces NF-κB phosphorylation and stimulates the expression of type I interferons and pro-inflammatory cytokines, including IL-1β, IL-6, and TNF-α. This cascade promotes the expression of VCAM-1, facilitating leukocyte adhesion and promoting endothelial dysfunction. Collectively, these molecular events bridge inflammatory signals with pro-coagulant processes, culminating in enhanced thrombus formation. Beyond its role in inflammation, eRNA significantly influences coagulation by interacting with TLR3 and inducing the expression of TF and proteases. These interactions augment the activation of coagulation factors XII and XI, key components of the “contact phase” of blood coagulation [109,110]. The eRNA–TLR3 axis has emerged as a promising therapeutic target in managing thrombotic disorders. RNase I treatment, which degrades circulating eRNA, has demonstrated efficacy in reducing the thrombus size and interrupting the inflammatory loop mediated by eRNA–TLR3 interactions. In general, RNase I administration significantly reduces the thrombus burden and neutrophil recruitment in murine models. Najem et al. observed that RNase I injection in rodents was associated with a significantly smaller thrombus size [76]. Treatment with RNase I also decreases fibrin deposition and TF activity [109,111].

A higher concentration of eRNA may explain hypercoagulable states in various pathological conditions. Conditions, such as sepsis, cancer, and severe tissue damage, are associated with increased extracellular RNA levels. This may lead to heightened thrombotic risks. Monitoring circulating RNA levels could serve as a diagnostic marker for thrombotic risk, and RNase-based therapies might mitigate these risks effectively.

These findings confirm RNase-based therapies as a potential antithrombotic strategy, disrupting the pro-coagulant and pro-inflammatory activities of eRNA. Higher concentrations of circulating eRNA may underlie hypercoagulable states observed in various pathological conditions, including cancer, sepsis, and tissue damage. These elevated eRNA levels may contribute to heightened thrombotic risks, making eRNA a potential diagnostic marker for thrombotic risk assessment (Figure 2). Monitoring circulating eRNA levels could provide insights into patient susceptibility to thrombotic events, while RNase-based therapies might effectively mitigate these risks. In conclusion, eRNA serves as a crucial mediator linking endothelial dysfunction, immune activation, and coagulation. Targeting eRNA pathways holds promise for the development of novel antithrombotic therapies, particularly in conditions characterized by excessive thromboinflammation.

## 9. MicroRNA as a Biomarker of Venous Thromboembolism

MicroRNAs (miRNAs) are small, non-coding, single-stranded RNA molecules, typically consisting of 22 nucleotides. They regulate gene expression by promoting the degradation of target messenger RNA (mRNA) or inhibiting its translation. miRNAs were first discovered in 1993 by Victor Ambros, and since then, they have emerged as crucial regulators of numerous cellular processes, offering promising potential for the development of novel therapeutic strategies [112,113].

In the context of hemostasis and thrombosis, miRNAs influence the expression of key hemostatic and fibrinolytic factors, thereby playing a critical role in thrombus formation and resolution. Specifically, miRNAs regulate hemostatic and pro-coagulant factors, affecting coagulation pathways and fibrinolysis inhibitors, modulating clot breakdown processes, as well as platelet biology, including platelet activation and aggregation, which are essential steps in thrombosis. Given their diverse roles, microRNAs have emerged as promising biomarkers for thrombotic diseases and potential therapeutic targets for modulating coagulation and the fibrinolytic balance [112]. By regulating the expression of key hemostatic factors and fibrinolytic components, miRNAs contribute significantly to thrombus formation and resolution. In the context of hemostasis and thrombosis, they influence clot dynamics by modulating pro-coagulant factors, fibrinolysis inhibitors, and other essential hemostatic regulators. Additionally, miRNAs play a critical role in platelet biology, impacting platelet activation and aggregation—processes central to thrombosis. Numerous studies using animal models of VTE have demonstrated that miRNA modulation can directly affect thrombus development and resolution. An elegant study by Sahu A et al. demonstrated the critical role of the *TF* gene in initiating the coagulation cascade [114]. The researchers showed that miR-145 directly binds to the 3′-UTR region of TF mRNA, inhibiting its expression. Consequently, reduced levels of miR-145 result in elevated TF expression, thereby promoting thrombogenesis [114]. Notably, the administration of miR-145 mimics in rats led to decreased TF levels and reduced thrombus formation. These mimics are chemically modified double-stranded RNA molecules designed to replicate endogenous miRNAs, downregulating target mRNA translation through sequestration or degradation. Conversely, treatment with miR-145 inhibitors increased TF activity and thrombus burden, further underscoring miR-145’s regulatory role. These findings suggest that the therapeutic restoration of miR-145 levels could prevent thrombosis without significant side effects. Supporting the translational relevance of these findings, venous thrombosis patients exhibited lower miR-145 levels and higher TF expression, mirroring the results observed in animal models. Overall, systemic miR-145 downregulation was associated with increased thrombus formation and TF expression, whereas treatment with miR-145 mimics effectively diminished both, highlighting the strong potential of miR-145-based therapies for managing venous thrombosis in humans [114].

The systemic downregulation of miR-145, coupled with the administration of miR-145 mimics, resulted in reduced TF expression and diminished thrombus formation. Similarly, miR-338-5p plays a crucial role in regulating IL-6 expression. As previously noted, IL-6 is a highly pro-inflammatory cytokine that contributes to thrombus formation [55]. Both in vitro studies and the intravenous administration of miR-338-5p have demonstrated a significant decrease in IL-6 expression and the thrombus burden [55]. In C57BL/6J mice with stenosis-induced VTE, elevated levels of miR-338-5p led to reduced IL-6 expression and decreased DVT formation, highlighting miR-338-5p as a potential therapeutic target.

Corroborating these findings, data from human VTE patients revealed significantly reduced miR-338-5p levels and a negative correlation between IL-6 and miR-338-5p expression [55]. These results suggest that restoring miR-338-5p levels may effectively mitigate DVT by downregulating IL-6. However, certain limitations must be considered. The study assumes that miR-338-5p alone can significantly regulate IL-6 expression in DVT, potentially overlooking the roles of other regulatory miRNAs or signaling molecules. Additionally, while animal models provide valuable insights, findings from mouse models may not always translate directly to humans due to interspecies physiological differences. Finally, the assumption that IL-6 is the primary relevant target of miR-338-5p in DVT warrants further investigation, as off-target effects could complicate therapeutic applications [55].

Several recent human studies have highlighted the potential role of microRNAs (miRNAs) as biomarkers for VTE in a clinical scenario. Patients with APE exhibited significantly higher levels of miR-134 compared to healthy controls and individuals diagnosed with cardiopulmonary diseases without acute PE [115]. Additionally, elevated levels of miR-195, miR-532, and miR-582 were observed in patients with provoked APE. These miRNAs were found at higher concentrations in VTE patients compared to healthy controls, further supporting their potential as diagnostic biomarkers [116]. MicroRNAs (miRNAs) may also serve as valuable biomarkers for distinguishing between patients with APE and ACS. Kessler et al. demonstrated that patients with APE exhibited higher serum concentrations of miR-1233 compared to controls [117]. Furthermore, miR-27a, miR-27b, miR-28-3p, miR-221, and miR-320b were found to be upregulated in patients with VTE compared to healthy controls [118]. Moreover, miR-27a and -b, 28-3p, miR-221, and miR-320b were upregulated in VTE patients as compared with controls [119,120,121]. In addition to their diagnostic potential, miRNAs regulate various stages of cellular pathways involved in disease pathogenesis, offering significant promise for targeted therapeutic interventions. However, several challenges continue to impede the translation of miRNA research into clinical applications for VTE, such as the limited sample size of studied cohorts and heterogenous study designs, including differences in sample collection, processing, and analysis methods, which significantly affect miRNA expression results, complicating cross-study comparisons. In addition, the interval between the thrombotic event and blood sampling influences circulating miRNA levels, potentially leading to variable results. To establish miRNAs as reliable biomarkers and therapeutic targets in VTE, large-scale studies with standardized methodologies are essential. A deeper understanding of how miRNAs contribute to VTE pathogenesis could pave the way for novel diagnostic tools and targeted therapeutic strategies aimed at improving patient outcomes.

## 10. Clinical Applications and Future Directions

Prompt and accurate diagnoses are essential to reduce the morbidity and mortality of APE. Traditional diagnostic approaches include clinical assessments, imaging modalities, like computed tomography pulmonary angiography, and laboratory biomarkers, such as D-dimer. While D-dimer is highly sensitive, its low specificity can lead to false positives and unnecessary imaging. Recent studies have highlighted the role of NETs in thrombosis, suggesting their potential as more specific biomarkers for PE diagnoses.

D-dimer is a fibrin-degradation product present in the blood after a blood clot dissolves. It is widely used in the diagnostic workup of suspected PE due to its high sensitivity, which allows clinicians to rule out PE when D-dimer levels are normal. However, elevated D-dimer levels are non-specific and can occur in various conditions, including infections, malignancies, trauma, pregnancy, and advanced age, leading to a high rate of false positives. This necessitates additional imaging studies to confirm PE, increasing healthcare costs and patient exposure to radiation.

The low specificity of D-dimer limits its utility as a standalone diagnostic tool for PE. Elevated levels can result from numerous non-thrombotic conditions, leading to unnecessary imaging and potential over-treatment. Therefore, there is a need for more specific biomarkers that can accurately identify thrombotic events like PE.

While D-dimer is highly sensitive for detecting thrombotic events, its low specificity limits its diagnostic accuracy. In contrast, NET biomarkers like H3Cit and MPO-DNA complexes have shown higher specificity for thrombosis [3,122]. For instance, NET biomarkers could complement D-dimer in diagnosing acute VTE, potentially reducing false positives and the need for unnecessary imaging.

Endothelium biomarkers may provide greater specificity for thrombotic events compared to D-dimer, reducing false-positive rates. NET biomarkers offer insights into the underlying mechanisms of thrombosis, potentially aiding in risk stratification and personalized treatment strategies. Combining NET biomarkers with D-dimer could enhance the diagnostic accuracy for PE, improving patient management and outcomes.

Incorporating NET biomarkers into existing diagnostic algorithms could improve the specificity of PE diagnoses. For example, measuring NET biomarkers alongside D-dimer in patients with suspected PE could help rule out false positives and reduce unnecessary imaging. This approach could be particularly beneficial in populations where D-dimer specificity is low, such as in elderly patients or those with comorbid conditions.

There is a need for standardized assays to accurately and reliably measure NET biomarkers. Large-scale prospective studies are required to validate the diagnostic performance of NET biomarkers in diverse patient populations. Integration into clinical workflows requires clear guidelines and a consideration of cost-effectiveness. Future research should focus on addressing these challenges and exploring the therapeutic potential of targeting NETs in thrombotic diseases. While D-dimer remains a valuable tool for ruling out PE due to its high sensitivity, its low specificity limits its standalone diagnostic utility. NET biomarkers, such as citrullinated histones and MPO–DNA complexes, have demonstrated higher specificity for thrombotic events and hold promise as complementary tools in diagnosing acute PE.

Incorporating NET biomarkers into diagnostic algorithms could enhance accuracy, reduce unnecessary imaging, and improve patient outcomes. However, further research is needed to standardize assays, validate findings, and establish clinical guidelines for their use.

miRNAs are small non-coding RNAs involved in gene regulation and are promising biomarkers because they are stable, detectable in body fluids, and accessible [117]. Several miRNAs (such as miR-126-3p, miR-222-3p, miR-27b-3p, and miR-451a) were found to be linked to VTE both in cancer and non-cancer settings. There is a need for larger, well-designed validation studies to develop an miRNA-based risk assessment model. The Khorana score, currently used for VTE risk prediction in cancer patients, underperforms in certain populations. miRNA-based models might outperform traditional scoring systems by improving the prediction accuracy. Differences in sample collection, analysis methods, and patient populations lead to inconsistencies in miRNA studies. More prospective cohort studies and validation in large populations are required before clinical applications. miRNAs hold significant potential as biomarkers for VTE risk prediction, but more validation studies are required before they can be used in clinical settings. There is a need to standardize approaches to analyze miRNA expression and combine miRNAs with current models for better diagnosis and prediction.

## 11. Summary

Our review highlights the relevant role of endothelial cells in mediating the interplay between thrombosis and inflammation. The process of EndMT and endothelial dysfunction serves as a key contributor to VTE and cardiovascular diseases. By understanding the underlying molecular mechanisms—particularly oxidative stress, cytokine signaling, and epigenetic regulation—potential new therapeutic strategies can be developed to prevent and treat VTE more effectively. In addition, miRNAs play a role in the regulation of the coagulation cascade and thrombus formation, making them promising biomarkers and therapeutic targets for VTE. Further translational studies, from bench to bedside, are needed to establish miRNA-based diagnostics and therapies for VTE.

## Figures and Tables

**Table 1:** Table showing, chronologically, the most relevant studies assessing inflammation in experimental models of acute pulmonary embolism.

Author	Year	Study Design	Conclusion
Eagleton et al. [12]	2002	Sprague–Dawley (SD) rats, IVC thrombosis-induced APE	Elevated macrophage infiltration and MCP-1 elevation within PA wall [12].
Tsang, J et al. [38]	2002	Piglets, thrombin-induced blood clots injected to the lower lobar PA	Minimal impact of IL-1β, Il-8, and TNF-α in systemic circulation [38].
Zagorski, J. et al. [13]	2003	Sprague–Dawley rats, polystyrene microsphere-induced APE	Rats with APE are characterized by severe pro-inflammatory chemokine accumulation in the lungs [13]
Watts, J.A. et al. [14]	2006	Sprague–Dawley rats, polystyrene microsphere-induced APE	Higher MCP-1 and MPO activity in damaged RV. Increased CINC-1, CINC-2, MIP-1α, and MCP-1 mRNA in damaged RV. Neutrophil and monocyte accumulation in RV among severe APE rats [14].
Zagorski et al. [15]	2007	Sprague–Dawley rats, polystyrene microsphere-induced APE	The crucial function of neutrophils in acute RV damage following APE episode. CINC-1 is responsible for neutrophil influx. Blockade with anti-PMN antibodies reduced RV dysfunction [15].
			Upregulated CCL-2, -3, -4, -6, -7, -9, -17, -20, and -27. Upregulated CXCL 1-2-9-19-16. Upregulated CCR1 and CXCR4. Downregulated CCL-12 and XCL-1 [15].
Fortuna, G.M. et al. [39]	2007	Mongrel dogs, micropshere-induced APE	MMP-9 mediated APE-induced pulmonary hypertension. Doxycycline treatment impaired APE-induced pulmonary hypertension [39].
Watts et al. [16]	2008	Sprague–Dawley rats, polystyrene microsphere-induced APE	The key role of macrophages and neutrophils in RV failure. The study revealed the presence of M1 cells in the early and M2 phenotype cells in the healing stage [16]. The neutrophil influx was diminished during 7 days following APE; monocyte influx was present for 6 weeks [16].
Dias-Junior, C.A. [40]	2009	Dogs, polystyrene microsphere-induced APE	NO–cGMP axis attenuated MMP-9 levels and reduced reactive oxygen species [40].
Zagorski et al. [18]	2009	Sprague–Dawley rats, polystyrene microsphere-induced APE	More exaggerated proinflammatory and profibrotic transcriptional response in the RV outflow tract compared with the apex [18].
Dolci, D.T. et al. [41]	2010	Pigs, polystyrene-induced APE	Higher bronchoalveolar lavage protein concentration was observed [41].
Tang, Z. et al. [19]	2016	Autologous thrombus-induced APE	The study applied an array to evaluate gene expression changes in the PA wall. Upregulated T- and B-cell, chemokine, NOD-like, Toll-like, RIG-I, and Fc-epsilon RI signaling pathways observed [19]. mRNA of IL-8 and TNF-α were increased in PA wall [19].
Zagórski, J. et al. [20]	2016	SD rats, microsphere-induced APE	Rats diagnosed with APE presented more exaggerated lung gene expression of inflammatory pathways and cholesterol synthesis, even without signs of pulmonary hypertension [20].
Wang, Y. et al. [42]	2020	Autologous thrombus-induced APE	Higher inflammatory cell infiltration, higher iNOS and higher IL-1β and IL-6, as well as TNF-α, mRNA were found in pulmonary and non-pulmonary parenchyma of rabbits with massive APE [42].

## Data Availability

No new data were created or analyzed in this study. Data sharing is not applicable to this article.

## Abbreviations

The following abbreviations are used in this manuscript:

APE	Acute pulmonary embolism
BAL	Bronchoalveolar lavage
CAM	Cell adhesion molecules
CINC	Cytokine-Induced Neutrophil Chemoattractant
CVD	Cardiovascular diseases
CXCL	Chemokine (C-X-C motif) ligand 1
DAMP	Danger associated molecular patterns
DVT	Deep vein thrombosis
EC	Endothelial cells
ED	Endothelium
F	Coagulation Factor
HMGB1	High mobility group box 1 protein
IL	Interleukin
IP-10	Interferon ɣ-induced protein 10
IVC	Inferior Vena Cava
MCP	Monocyte chemoattractant protein
MIP	Macrophage Inflammatory Protein
miRNA	Micro-RNA
MMP	Matrix metalloproteinases
MPO	Myeloperoxidase
NETs	Neutrophils extracellular traps
NO	Nitric oxide
PA	Pulmonary Artery
PTS	Post-thrombotic syndrome
RAGE	Receptor for advanced glycation end products
ROS	Reactive Oxygen Species
RV	Right ventricle
RVOT	Right ventricle outflow tract
RVSP	Right ventricle systolic pressure
SNP	Single nucleotide polymorphism
TF	Tissue factor
TFPI	Tissue factor plasminogen inhibitor
TIMP	Tissue inhibitor of metalloproteinases
TLR	Toll like receptor
TM	Thrombomodulin
TNF-α	Tumor necrosis factor α
tPA	Tissue Plasminogen Activator
uPA	Urokinase plasminogen activator
WPB	Weibel Palade Bodies
VTE	Venous Thromboembolism
VWF	Von Willebrandt Factor

## References

[1] Engelmann B., Massberg S. (2013). Thrombosis as an intravascular effector of innate immunity. Nat. Rev. Immunol..

[2] Marcos-Jubilar M., Lecumberri R., Paramo J.A. (2023). Immunothrombosis: Molecular Aspects and New Therapeutic Perspectives. J. Clin. Med..

[3] Zabczyk M., Natorska J., Undas A. (2024). Novel factors affecting fibrin clot formation and their clinical implications. Pol. Arch. Intern. Med..

[4] Pajak A., Jankowski P., Zdrojewski T. (2022). The burden of cardiovascular disease risk factors: A current problem. Kardiol. Pol..

[5] Vaduganathan M., Mensah G.A., Turco J.V., Fuster V., Roth G.A. (2022). The Global Burden of Cardiovascular Diseases and Risk: A Compass for Future Health. J. Am. Coll. Cardiol..

[6] Silva B.V., Cale R., Menezes M.N., Jorge C., Pinto F.J., Caldeira D. (2023). How to predict prognosis in patients with acute pulmonary embolism? Recent advances. Kardiol. Pol..

[7] Page I.H. (1949). Pathogenesis of arterial hypertension. J. Am. Med. Assoc..

[8] Imiela A.M., Mikolajczyk T.P., Guzik T.J., Pruszczyk P. (2024). Acute Pulmonary Embolism and Immunity in Animal Models. Arch. Immunol. Ther. Exp..

[9] Imiela A.M., Mikolajczyk T.P., Pruszczyk P. (2024). Novel Insight into Inflammatory Pathways in Acute Pulmonary Embolism in Humans. Arch. Immunol. Ther. Exp..

[10] Ridker P.M., Everett B.M., Thuren T., MacFadyen J.G., Chang W.H., Ballantyne C., Fonseca F., Nicolau J., Koenig W., Anker S.D. (2017). Antiinflammatory Therapy with Canakinumab for Atherosclerotic Disease. N. Engl. J. Med..

[11] Tardif J.C., Kouz S., Waters D.D., Bertrand O.F., Diaz R., Maggioni A.P., Pinto F.J., Ibrahim R., Gamra H., Kiwan G.S. (2019). Efficacy and Safety of Low-Dose Colchicine after Myocardial Infarction. N. Engl. J. Med..

[12] Eagleton M.J., Henke P.K., Luke C.E., Hawley A.E., Bedi A., Knipp B.S., Wakefield T.W., Greenfield L.J. (2002). Southern Association for Vascular Surgery William J. von Leibig Award. Inflammation and intimal hyperplasia associated with experimental pulmonary embolism. J. Vasc. Surg..

[13] Zagorski J., Debelak J., Gellar M., Watts J.A., Kline J.A. (2003). Chemokines accumulate in the lungs of rats with severe pulmonary embolism induced by polystyrene microspheres. J. Immunol..

[14] Watts J.A., Zagorski J., Gellar M.A., Stevinson B.G., Kline J.A. (2006). Cardiac inflammation contributes to right ventricular dysfunction following experimental pulmonary embolism in rats. J. Mol. Cell. Cardiol..

[15] Zagorski J., Gellar M.A., Obraztsova M., Kline J.A., Watts J.A. (2007). Inhibition of CINC-1 decreases right ventricular damage caused by experimental pulmonary embolism in rats. J. Immunol..

[16] Watts J.A., Gellar M.A., Obraztsova M., Kline J.A., Zagorski J. (2008). Role of inflammation in right ventricular damage and repair following experimental pulmonary embolism in rats. Int. J. Exp. Pathol..

[17] Zagorski J., Sanapareddy N., Gellar M.A., Kline J.A., Watts J.A. (2008). Transcriptional profile of right ventricular tissue during acute pulmonary embolism in rats. Physiol. Genom..

[18] Zagorski J., Obraztsova M., Gellar M.A., Kline J.A., Watts J.A. (2009). Transcriptional changes in right ventricular tissues are enriched in the outflow tract compared with the apex during chronic pulmonary embolism in rats. Physiol. Genom..

[19] Tang Z., Wang X., Huang J., Zhou X., Xie H., Zhu Q., Huang M., Ni S. (2016). Gene Expression Profiling of Pulmonary Artery in a Rabbit Model of Pulmonary Thromboembolism. PLoS ONE.

[20] Zagorski J., Kline J.A. (2016). Differential effect of mild and severe pulmonary embolism on the rat lung transcriptome. Respir. Res..

[21] Colling M.E., Tourdot B.E., Kanthi Y. (2021). Inflammation, Infection and Venous Thromboembolism. Circ. Res..

[22] Semeraro F., Ammollo C.T., Semeraro N., Colucci M. (2009). Tissue factor-expressing monocytes inhibit fibrinolysis through a TAFI-mediated mechanism, and make clots resistant to heparins. Haematologica.

[23] Preston R.J.S., O’Sullivan J.M., O’Donnell J.S. (2019). Advances in understanding the molecular mechanisms of venous thrombosis. Br. J. Haematol..

[24] von Bruhl M.L., Stark K., Steinhart A., Chandraratne S., Konrad I., Lorenz M., Khandoga A., Tirniceriu A., Coletti R., Kollnberger M. (2012). Monocytes, neutrophils, and platelets cooperate to initiate and propagate venous thrombosis in mice in vivo. J. Exp. Med..

[25] Kimball A.S., Obi A.T., Luke C.E., Dowling A.R., Cai Q., Adili R., Jankowski H., Schaller M., Holinstadt M., Jaffer F.A. (2020). Ly6CLo Monocyte/Macrophages are Essential for Thrombus Resolution in a Murine Model of Venous Thrombosis. Thromb. Haemost..

[26] Uderhardt S., Ackermann J.A., Fillep T., Hammond V.J., Willeit J., Santer P., Mayr M., Biburger M., Miller M., Zellner K.R. (2017). Enzymatic lipid oxidation by eosinophils propagates coagulation, hemostasis, and thrombotic disease. J. Exp. Med..

[27] Natorska J., Zabczyk M., Undas A. (2023). Neutrophil extracellular traps (NETs) in cardiovascular diseases: From molecular mechanisms to therapeutic interventions. Kardiol. Pol..

[28] Brinkmann V., Reichard U., Goosmann C., Fauler B., Uhlemann Y., Weiss D.S., Weinrauch Y., Zychlinsky A. (2004). Neutrophil extracellular traps kill bacteria. Science.

[29] Lewis H.D., Liddle J., Coote J.E., Atkinson S.J., Barker M.D., Bax B.D., Bicker K.L., Bingham R.P., Campbell M., Chen Y.H. (2015). Inhibition of PAD4 activity is sufficient to disrupt mouse and human NET formation. Nat. Chem. Biol..

[30] Simon D.I., Chen Z., Xu H., Li C.Q., Dong J., McIntire L.V., Ballantyne C.M., Zhang L., Furman M.I., Berndt M.C. (2000). Platelet glycoprotein ibalpha is a counterreceptor for the leukocyte integrin Mac-1 (CD11b/CD18). J. Exp. Med..

[31] Lam F.W., Cruz M.A., Parikh K., Rumbaut R.E. (2016). Histones stimulate von Willebrand factor release in vitro and in vivo. Haematologica.

[32] Ammollo C.T., Semeraro F., Xu J., Esmon N.L., Esmon C.T. (2011). Extracellular histones increase plasma thrombin generation by impairing thrombomodulin-dependent protein C activation. J. Thromb. Haemost..

[33] Brill A., Fuchs T.A., Savchenko A.S., Thomas G.M., Martinod K., De Meyer S.F., Bhandari A.A., Wagner D.D. (2012). Neutrophil extracellular traps promote deep vein thrombosis in mice. J. Thromb. Haemost..

[34] Massberg S., Grahl L., von Bruehl M.L., Manukyan D., Pfeiler S., Goosmann C., Brinkmann V., Lorenz M., Bidzhekov K., Khandagale A.B. (2010). Reciprocal coupling of coagulation and innate immunity via neutrophil serine proteases. Nat. Med..

[35] Schulman S., Makatsariya A., Khizroeva J., Bitsadze V., Kapanadze D. (2024). The Basic Principles of Pathophysiology of Venous Thrombosis. Int. J. Mol. Sci..

[36] Smith P., Rosell A., Farm M., Bruzelius M., Aguilera Gatica K., Mackman N., Odeberg J., Thalin C. (2022). Markers of neutrophil activation and neutrophil extracellular traps in diagnosing patients with acute venous thromboembolism: A feasibility study based on two VTE cohorts. PLoS ONE.

[37] Mosevoll K.A., Johansen S., Wendelbo O., Nepstad I., Bruserud O., Reikvam H. (2018). Cytokines, Adhesion Molecules, and Matrix Metalloproteases as Predisposing, Diagnostic, and Prognostic Factors in Venous Thrombosis. Front. Med..

[38] Tsang J., Simon M., Stewart K., Qayumi K., Battistini B. (2002). Proinflammatory cytokines are not released in the circulation following acute pulmonary thromboembolism in pigs. J. Investig. Surg..

[39] Fortuna G.M., Figueiredo-Lopes L., Dias-Junior C.A., Gerlach R.F., Tanus-Santos J.E. (2007). A role for matrix metalloproteinase-9 in the hemodynamic changes following acute pulmonary embolism. Int. J. Cardiol..

[40] Dias-Junior C.A., Cau S.B., Oliveira A.M., Castro M.M., Montenegro M.F., Gerlach R.F., Tanus-Santos J.E. (2009). Nitrite or sildenafil, but not BAY 41-2272, blunt acute pulmonary embolism-induced increases in circulating matrix metalloproteinase-9 and oxidative stress. Thromb. Res..

[41] Dolci D.T., Fuentes C.B., Rolim D., Park M., Schettino G.P., Azevedo L.C. (2010). Time course of haemodynamic, respiratory and inflammatory disturbances induced by experimental acute pulmonary polystyrene microembolism. Eur. J. Anaesthesiol..

[42] Wang Y., Yu D., Yu Y., Liu X., Hu L., Gu Y. (2020). Association Between Inflammatory Mediators and Pulmonary Blood Flow in a Rabbit Model of Acute Pulmonary Embolism Combined With Shock. Front. Physiol..

[43] van Minkelen R., de Visser M.C., Houwing-Duistermaat J.J., Vos H.L., Bertina R.M., Rosendaal F.R. (2007). Haplotypes of IL1B, IL1RN, IL1R1, and IL1R2 and the risk of venous thrombosis. Arterioscler. Thromb. Vasc. Biol..

[44] Malaponte G., Polesel J., Candido S., Sambataro D., Bevelacqua V., Anzaldi M., Vella N., Fiore V., Militello L., Mazzarino M.C. (2013). IL-6-174 G>C and MMP-9-1562 C>T polymorphisms are associated with increased risk of deep vein thrombosis in cancer patients. Cytokine.

[45] Nosaka M., Ishida Y., Kimura A., Kuninaka Y., Inui M., Mukaida N., Kondo T. (2011). Absence of IFN-gamma accelerates thrombus resolution through enhanced MMP-9 and VEGF expression in mice. J. Clin. Investig..

[46] Henke P.K. (2007). Plasmin and matrix metalloproteinase system in deep venous thrombosis resolution. Vascular.

[47] de Franciscis S., Gallelli L., Amato B., Butrico L., Rossi A., Buffone G., Calio F.G., De Caridi G., Grande R., Serra R. (2016). Plasma MMP and TIMP evaluation in patients with deep venous thrombosis: Could they have a predictive role in the development of post-thrombotic syndrome?. Int. Wound J..

[48] Abuduhalike R., Sun J., Mahemuti A. (2020). Correlation Study of the Long-Term Prognosis of Venous Thromboembolism and Inflammatory Gene Polymorphisms. Int. J. Gen. Med..

[49] van Aken B.E., den Heijer M., Bos G.M., van Deventer S.J., Reitsma P.H. (2000). Recurrent venous thrombosis and markers of inflammation. Thromb. Haemost..

[50] Roumen-Klappe E.M., den Heijer M., van Uum S.H., van der Ven-Jongekrijg J., van der Graaf F., Wollersheim H. (2002). Inflammatory response in the acute phase of deep vein thrombosis. J. Vasc. Surg..

[51] Roumen-Klappe E.M., Janssen M.C., Van Rossum J., Holewijn S., Van Bokhoven M.M., Kaasjager K., Wollersheim H., Den Heijer M. (2009). Inflammation in deep vein thrombosis and the development of post-thrombotic syndrome: A prospective study. J. Thromb. Haemost..

[52] Beckers M.M., Ruven H.J., Haas F.J., Doevendans P.A., ten Cate H., Prins M.H., Biesma D.H. (2010). Single nucleotide polymorphisms in inflammation-related genes are associated with venous thromboembolism. Eur. J. Intern. Med..

[53] Matos M.F., Lourenco D.M., Orikaza C.M., Bajerl J.A., Noguti M.A., Morelli V.M. (2011). The role of IL-6, IL-8 and MCP-1 and their promoter polymorphisms IL-6-174GC, IL-8-251AT and MCP-1-2518AG in the risk of venous thromboembolism: A case-control study. Thromb. Res..

[54] Bittar L.F., Mazetto Bde M., Orsi F.L., Collela M.P., De Paula E.V., Annichino-Bizzacchi J.M. (2015). Long-term increased factor VIII levels are associated to interleukin-6 levels but not to post-thrombotic syndrome in patients with deep venous thrombosis. Thromb. Res..

[55] Zhang Y., Zhang Z., Wei R., Miao X., Sun S., Liang G., Chu C., Zhao L., Zhu X., Guo Q. (2020). IL (Interleukin)-6 Contributes to Deep Vein Thrombosis and Is Negatively Regulated by miR-338-5p. Arterioscler. Thromb. Vasc. Biol..

[56] van Aken B.E., Reitsma P.H., Rosendaal F.R. (2002). Interleukin 8 and venous thrombosis: Evidence for a role of inflammation in thrombosis. Br. J. Haematol..

[57] Montes-Worboys A., Arellano E., Elias T., Leon J., Rodriguez-Portal J.A., Otero R. (2013). Residual thrombosis after a first episode of proximal deep venous thrombosis. Blood Coagul. Fibrinolysis.

[58] Bontekoe E., Brailovsky Y., Hoppensteadt D., Bontekoe J., Siddiqui F., Newman J., Iqbal O., Reed T., Fareed J., Darki A. (2021). Upregulation of Inflammatory Cytokines in Pulmonary Embolism Using Biochip-Array Profiling. Clin. Appl. Thromb./Hemost..

[59] Horakova K., Chylkova A., Kolorz M., Bartosova L., Pechacek V., Starostka D., Wroblova K. (2012). Polymorphism G-308A in the promoter of the tumor necrosis factor-alpha gene and its association with the risk of venous thromboembolism. Blood Coagul. Fibrinolysis.

[60] Mazetto B.M., Orsi F.L., Barnabe A., De Paula E.V., Flores-Nascimento M.C., Annichino-Bizzacchi J.M. (2012). Increased ADAMTS13 activity in patients with venous thromboembolism. Thromb. Res..

[61] Memon A.A., Sundquist K., Wang X., Svensson P.J., Sundquist J., Zoller B. (2014). Transforming growth factor (TGF)-beta levels and unprovoked recurrent venous thromboembolism. J. Thromb. Thrombolysis.

[62] Wang X., Sundquist K., Svensson P.J., Rastkhani H., Palmer K., Memon A.A., Sundquist J., Zoller B. (2019). Association of recurrent venous thromboembolism and circulating microRNAs. Clin. Epigenet..

[63] Shbaklo H., Holcroft C.A., Kahn S.R. (2009). Levels of inflammatory markers and the development of the post-thrombotic syndrome. Thromb. Haemost..

[64] Vandy F.C., Stabler C., Eliassen A.M., Hawley A.E., Guire K.E., Myers D.D., Henke P.K., Wakefield T.W. (2013). Soluble P-selectin for the diagnosis of lower extremity deep venous thrombosis. J. Vasc. Surg. Venous Lymphat. Disord..

[65] Pilard M., Ollivier E.L., Gourdou-Latyszenok V., Couturaud F., Lemarie C.A. (2022). Endothelial Cell Phenotype, a Major Determinant of Venous Thrombo-Inflammation. Front. Cardiovasc. Med..

[66] Imiela A.M., Mikolajczyk T.P., Siedlinski M., Dobrowolski P., Konior-Rozlachowska A., Wrobel A., Biernat-Kaluza E., Januszewicz M., Guzik B., Guzik T.J. (2022). Th17/Treg imbalance in patients with primary hyperaldosteronism and resistant hypertension. Pol. Arch. Intern. Med..

[67] Imiela A.M., Siedlinski M., Dobrowolski P., Pregowska-Chwala B., Kabat M., Oliveira Silva R.N., Koshy A.M., Wrobel A., Cendrowska-Demkow I., Januszewicz M. (2022). Altered monocytic phenotypes are linked to a hypertension form: A clinical observational study. Kardiol. Pol..

[68] Itani H.A., McMaster W.G., Saleh M.A., Nazarewicz R.R., Mikolajczyk T.P., Kaszuba A.M., Konior A., Prejbisz A., Januszewicz A., Norlander A.E. (2016). Activation of Human T Cells in Hypertension: Studies of Humanized Mice and Hypertensive Humans. Hypertension.

[69] Biernat K., Kuciel N., Mazurek J., Hap K. (2024). Is It Possible to Train the Endothelium?-A Narrative Literature Review. Life.

[70] Peracaula M., Sebastian L., Francisco I., Vilaplana M.B., Rodriguez-Chiaradia D.A., Tura-Ceide O. (2024). Decoding Pulmonary Embolism: Pathophysiology, Diagnosis, and Treatment. Biomedicines.

[71] Pacinella G., Ciaccio A.M., Tuttolomondo A. (2022). Endothelial Dysfunction and Chronic Inflammation: The Cornerstones of Vascular Alterations in Age-Related Diseases. Int. J. Mol. Sci..

[72] Shirinsky V.P. (2024). Vascular Endothelium at the Molecular Level: From Fundamental Knowledge Toward Medical Implementation. Biomedicines.

[73] El-Mansi S., Nightingale T.D. (2021). Emerging mechanisms to modulate VWF release from endothelial cells. Int. J. Biochem. Cell Biol..

[74] Zindel J., Kubes P. (2020). DAMPs, PAMPs, and LAMPs in Immunity and Sterile Inflammation. Annu. Rev. Pathol..

[75] Gong T., Liu L., Jiang W., Zhou R. (2020). DAMP-sensing receptors in sterile inflammation and inflammatory diseases. Nat. Rev. Immunol..

[76] Najem M.Y., Couturaud F., Lemarie C.A. (2020). Cytokine and chemokine regulation of venous thromboembolism. J. Thromb. Haemost..

[77] Iba T., Levy J.H. (2018). Inflammation and thrombosis: Roles of neutrophils, platelets and endothelial cells and their interactions in thrombus formation during sepsis. J. Thromb. Haemost..

[78] Laurance S., Bertin F.R., Ebrahimian T., Kassim Y., Rys R.N., Lehoux S., Lemarie C.A., Blostein M.D. (2017). Gas6 Promotes Inflammatory (CCR2(hi)CX3CR1(lo)) Monocyte Recruitment in Venous Thrombosis. Arterioscler. Thromb. Vasc. Biol..

[79] Kaiser R., Escaig R., Erber J., Nicolai L. (2021). Neutrophil-Platelet Interactions as Novel Treatment Targets in Cardiovascular Disease. Front. Cardiovasc. Med..

[80] Ramacciotti E., Blackburn S., Hawley A.E., Vandy F., Ballard-Lipka N., Stabler C., Baker N., Guire K.E., Rectenwald J.E., Henke P.K. (2011). Evaluation of soluble P-selectin as a marker for the diagnosis of deep venous thrombosis. Clin. Appl. Thromb./Hemost..

[81] Donadini M.P., Calcaterra F., Romualdi E., Ciceri R., Cancellara A., Lodigiani C., Bacci M., Della Bella S., Ageno W., Mavilio D. (2025). The Link Between Venous and Arterial Thrombosis: Is There a Role for Endothelial Dysfunction?. Cells.

[82] Dzikowska-Diduch O., Domienik-Karlowicz J., Gorska E., Demkow U., Pruszczyk P., Kostrubiec M. (2017). E-selectin and sICAM-1, biomarkers of endothelial function, predict recurrence of venous thromboembolism. Thromb. Res..

[83] Purdy M., Obi A., Myers D., Wakefield T. (2022). P- and E- selectin in venous thrombosis and non-venous pathologies. J. Thromb. Haemost..

[84] Darwish I., Fareed J., Brailovsky Y., Hoppensteadt D., Slajus B., Bontekoe E., De Stefano F., Reed T., Darki A. (2022). Dysregulation of Biomarkers of Hemostatic Activation and Inflammatory Processes are Associated with Adverse Outcomes in Pulmonary Embolism. Clin. Appl. Thromb./Hemost..

[85] McCormack J.J., Lopes da Silva M., Ferraro F., Patella F., Cutler D.F. (2017). Weibel-Palade bodies at a glance. J. Cell Sci..

[86] Brill A., Fuchs T.A., Chauhan A.K., Yang J.J., De Meyer S.F., Kollnberger M., Wakefield T.W., Lammle B., Massberg S., Wagner D.D. (2011). von Willebrand factor-mediated platelet adhesion is critical for deep vein thrombosis in mouse models. Blood.

[87] Dong R., Chen W., Feng W., Xia C., Hu D., Zhang Y., Yang Y., Wang D.W., Xu X., Tu L. (2015). Exogenous Bradykinin Inhibits Tissue Factor Induction and Deep Vein Thrombosis via Activating the eNOS/Phosphoinositide 3-Kinase/Akt Signaling Pathway. Cell. Physiol. Biochem..

[88] Ghasemzadeh M., Hosseini E. (2015). Intravascular leukocyte migration through platelet thrombi: Directing leukocytes to sites of vascular injury. Thromb. Haemost..

[89] Suarez-Rivero J.M., Pastor-Maldonado C.J., Povea-Cabello S., Alvarez-Cordoba M., Villalon-Garcia I., Talaveron-Rey M., Suarez-Carrillo A., Munuera-Cabeza M., Sanchez-Alcazar J.A. (2021). From Mitochondria to Atherosclerosis: The Inflammation Path. Biomedicines.

[90] Fiuza C., Bustin M., Talwar S., Tropea M., Gerstenberger E., Shelhamer J.H., Suffredini A.F. (2003). Inflammation-promoting activity of HMGB1 on human microvascular endothelial cells. Blood.

[91] Venereau E., Schiraldi M., Uguccioni M., Bianchi M.E. (2013). HMGB1 and leukocyte migration during trauma and sterile inflammation. Mol. Immunol..

[92] Stark K., Philippi V., Stockhausen S., Busse J., Antonelli A., Miller M., Schubert I., Hoseinpour P., Chandraratne S., von Bruhl M.L. (2016). Disulfide HMGB1 derived from platelets coordinates venous thrombosis in mice. Blood.

[93] Dyer M.R., Chen Q., Haldeman S., Yazdani H., Hoffman R., Loughran P., Tsung A., Zuckerbraun B.S., Simmons R.L., Neal M.D. (2018). Deep vein thrombosis in mice is regulated by platelet HMGB1 through release of neutrophil-extracellular traps and DNA. Sci. Rep..

[94] Vogel S., Bodenstein R., Chen Q., Feil S., Feil R., Rheinlaender J., Schaffer T.E., Bohn E., Frick J.S., Borst O. (2015). Platelet-derived HMGB1 is a critical mediator of thrombosis. J. Clin. Investig..

[95] Choi H.W., Tian M., Song F., Venereau E., Preti A., Park S.W., Hamilton K., Swapna G.V., Manohar M., Moreau M. (2015). Aspirin’s Active Metabolite Salicylic Acid Targets High Mobility Group Box 1 to Modulate Inflammatory Responses. Mol. Med..

[96] Souilhol C., Harmsen M.C., Evans P.C., Krenning G. (2018). Endothelial-mesenchymal transition in atherosclerosis. Cardiovasc. Res..

[97] Kovacic J.C., Dimmeler S., Harvey R.P., Finkel T., Aikawa E., Krenning G., Baker A.H. (2019). Endothelial to Mesenchymal Transition in Cardiovascular Disease: JACC State-of-the-Art Review. J. Am. Coll. Cardiol..

[98] Song S., Zhang R., Cao W., Fang G., Yu Y., Wan Y., Wang C., Li Y., Wang Q. (2019). Foxm1 is a critical driver of TGF-beta-induced EndMT in endothelial cells through Smad2/3 and binds to the Snail promoter. J. Cell. Physiol..

[99] Yun E., Kook Y., Yoo K.H., Kim K.I., Lee M.S., Kim J., Lee A. (2020). Endothelial to Mesenchymal Transition in Pulmonary Vascular Diseases. Biomedicines.

[100] Jackson A.O., Zhang J., Jiang Z., Yin K. (2017). Endothelial-to-mesenchymal transition: A novel therapeutic target for cardiovascular diseases. Trends Cardiovasc. Med..

[101] Shu D.Y., Butcher E., Saint-Geniez M. (2020). EMT and EndMT: Emerging Roles in Age-Related Macular Degeneration. Int. J. Mol. Sci..

[102] Gorelova A., Berman M., Al Ghouleh I. (2021). Endothelial-to-Mesenchymal Transition in Pulmonary Arterial Hypertension. Antioxid. Redox Signal..

[103] Evrard S.M., Lecce L., Michelis K.C., Nomura-Kitabayashi A., Pandey G., Purushothaman K.R., d’Escamard V., Li J.R., Hadri L., Fujitani K. (2016). Endothelial to mesenchymal transition is common in atherosclerotic lesions and is associated with plaque instability. Nat. Commun..

[104] Peng Q., Shan D., Cui K., Li K., Zhu B., Wu H., Wang B., Wong S., Norton V., Dong Y. (2022). The Role of Endothelial-to-Mesenchymal Transition in Cardiovascular Disease. Cells.

[105] Terriaca S., Ferlosio A., Scioli M.G., Coppa F., Bertoldo F., Pisano C., Belmonte B., Balistreri C.R., Orlandi A. (2024). miRNA Regulation of Cell Phenotype and Parietal Remodeling in Atherosclerotic and Non-Atherosclerotic Aortic Aneurysms: Differences and Similarities. Int. J. Mol. Sci..

[106] Jayasuriya R., Ganesan K., Xu B., Ramkumar K.M. (2022). Emerging role of long non-coding RNAs in endothelial dysfunction and their molecular mechanisms. Biomed. Pharmacother..

[107] Nishita-Hiresha V., Varsha R., Jayasuriya R., Ramkumar K.M. (2023). The role of circRNA-miRNA-mRNA interaction network in endothelial dysfunction. Gene.

[108] Kannemeier C., Shibamiya A., Nakazawa F., Trusheim H., Ruppert C., Markart P., Song Y., Tzima E., Kennerknecht E., Niepmann M. (2007). Extracellular RNA constitutes a natural procoagulant cofactor in blood coagulation. Proc. Natl. Acad. Sci. USA.

[109] Savino L., Savino M., Kansakar U., Dazzetti T., Varzideh F., Jankauskas S.S., Mone P., Santulli G. (2024). Extracellular RNA and Endothelial TLR3 Link Inflammation and Venous Thromboembolism. J. Am. Heart Assoc..

[110] Lind N.A., Rael V.E., Pestal K., Liu B., Barton G.M. (2022). Regulation of the nucleic acid-sensing Toll-like receptors. Nat. Rev. Immunol..

[111] Bhagat S., Biswas I., Ahmed R., Khan G.A. (2020). Hypoxia induced up-regulation of tissue factor is mediated through extracellular RNA activated Toll-like receptor 3-activated protein 1 signalling. Blood Cells Mol. Dis..

[112] Morelli V.M., Braekkan S.K., Hansen J.B. (2020). Role of microRNAs in Venous Thromboembolism. Int. J. Mol. Sci..

[113] Saliminejad K., Khorram Khorshid H.R., Soleymani Fard S., Ghaffari S.H. (2019). An overview of microRNAs: Biology, functions, therapeutics, and analysis methods. J. Cell. Physiol..

[114] Sahu A., Jha P.K., Prabhakar A., Singh H.D., Gupta N., Chatterjee T., Tyagi T., Sharma S., Kumari B., Singh S. (2017). MicroRNA-145 Impedes Thrombus Formation via Targeting Tissue Factor in Venous Thrombosis. EBioMedicine.

[115] Xiao J., Jing Z.C., Ellinor P.T., Liang D., Zhang H., Liu Y., Chen X., Pan L., Lyon R., Liu Y. (2011). MicroRNA-134 as a potential plasma biomarker for the diagnosis of acute pulmonary embolism. J. Transl. Med..

[116] Qin J., Liang H., Shi D., Dai J., Xu Z., Chen D., Chen X., Jiang Q. (2015). A panel of microRNAs as a new biomarkers for the detection of deep vein thrombosis. J. Thromb. Thrombolysis.

[117] Anijs R.J.S., Nguyen Y.N., Cannegieter S.C., Versteeg H.H., Buijs J.T. (2023). MicroRNAs as prognostic biomarkers for (cancer-associated) venous thromboembolism. J. Thromb. Haemost..

[118] Kessler T., Erdmann J., Vilne B., Bruse P., Kurowski V., Diemert P., Schunkert H., Sager H.B. (2016). Serum microRNA-1233 is a specific biomarker for diagnosing acute pulmonary embolism. J. Transl. Med..

[119] Liu T., Kang J., Liu F. (2018). Plasma Levels of microRNA-221 (miR-221) are Increased in Patients with Acute Pulmonary Embolism. Med. Sci. Monit..

[120] Wang Q., Ma J., Jiang Z., Wu F., Ping J., Ming L. (2018). Diagnostic value of circulating microRNA-27a/b in patients with acute pulmonary embolism. Int. Angiol..

[121] Jiang Z., Ma J., Wang Q., Wu F., Ping J., Ming L. (2018). Combination of Circulating miRNA-320a/b and D-Dimer Improves Diagnostic Accuracy in Deep Vein Thrombosis Patients. Med. Sci. Monit..

[122] Liu F., Zhai Q. (2024). Expression level of neutrophil extracellular traps in peripheral blood of patients with chronic heart failure complicated with venous thrombosis and its clinical significance. J. Cardiothorac. Surg..

